# Discovery and validation of immune-associated long non-coding RNA biomarkers associated with clinically molecular subtype and prognosis in diffuse large B cell lymphoma

**DOI:** 10.1186/s12943-017-0580-4

**Published:** 2017-01-19

**Authors:** Meng Zhou, Hengqiang Zhao, Wanying Xu, Siqi Bao, Liang Cheng, Jie Sun

**Affiliations:** College of Bioinformatics Science and Technology, Harbin Medical University, Harbin, 150081 People’s Republic of China

**Keywords:** Biomarkers, Subtype classification, Diffuse large B-cell lymphoma, Long non-coding RNAs, Prognosis

## Abstract

**Background:**

Diffuse large B-cell lymphoma (DLBCL) is an aggressive and complex disease characterized by wide clinical, phenotypic and molecular heterogeneities. The expression pattern and clinical implication of long non-coding RNAs (lncRNAs) between germinal center B-cell-like (GCB) and activated B-cell-like (ABC) subtypes in DLBCL remain unclear. This study aims to determine whether lncRNA can serve as predictive biomarkers for subtype classification and prognosis in DLBCL.

**Methods:**

Genome-wide comparative analysis of lncRNA expression profiles were performed in a large number of DLBCL patients from Gene Expression Omnibus (GEO), including GSE31312 cohort (*N* = 426), GSE10846 (*N* = 350) cohort and GSE4475 cohort (*N* = 129). Novel lncRNA biomarkers associated with clinically molecular subtype and prognosis were identified in the discovery cohort using differential expression analyses and weighted voting algorithm. The predictive value of the lncRNA signature was then assessed in two independent cohorts. The functional implication of lncRNA signature was also analyzed by integrative analysis of lncRNA and mRNA.

**Results:**

Seventeen of the 156 differentially expressed lncRNAs between GCB and ABC subtypes were identified as candidate biomarkers and integrated into form a lncRNA-based signature (termed SubSigLnc-17) which was able to discriminate between GCB and ABC subtypes with AUC of 0.974, specificity of 89.6% and sensitivity of 92.5%. Furthermore, subgroups of patients characterized by the SubSigLnc-17 demonstrated significantly different clinical outcome. The reproducible predictive power of SubSigLnc-17 in subtype classification and prognosis was successfully validated in the internal validation cohort and another two independent patient cohorts. Integrative analysis of lncRNA-mRNA suggested that these candidate lncRNA biomarkers were mainly related to immune-associated processes, such as T cell activation, leukocyte activation, lymphocyte activation and Chemokine signaling pathway.

**Conclusions:**

Our study uncovered differentiated lncRNA expression pattern between GCB and ABC DLBCL and identified a 17-lncRNA signature for subtype classification and prognosis prediction. With further prospective validation, our study will improve the understanding of underlying molecular heterogeneities in DLBCL and provide candidate lncRNA biomarkers in DLBCL classification and prognosis.

**Electronic supplementary material:**

The online version of this article (doi:10.1186/s12943-017-0580-4) contains supplementary material, which is available to authorized users.

## Background

Diffuse large B-cell lymphoma (DLBCL) occurs most commonly in all subtypes of non-Hodgkin lymphoma (NHL), representing more than one-third of all diagnosed NHL cases and making it the most prevalent form of NHL among adults worldwide [1, 2]. Evidence from biological and clinical studies demonstrated that DLBCL is an aggressive and complex disease characterized by wide clinical, phenotypic and molecular heterogeneities [3–5]. Although the survival rate has improved dramatically and could reach 50% ~ 60%, heterogeneity properties of DLBCL contributed to different clinical outcome for DLBCL patients with current standard therapy (Rituximab combined with traditional chemotherapy of cyclophosphamide-doxorubicin-vincristine-prednisone (R-CHOP)) [6]. With the emergence of high-throughput technologies, two major molecular subtypes first were identified by microarray-based gene expression profiling on the basis of gene expression pattern: germinal center B-cell-like (GCB) and activated B-cell-like (ABC) [7]. The distinct prognostic implications of these molecular subtypes have also been observed: patients with GCB DLBCL exhibited more favorable clinical outcome with 5-year progression-free survival (PFS) of 73% than those with ABC DLBCL with 5-year PFS of 48% following R-CHOP therapy [6]. Several groups have identified mRNA or miRNA-focus prognostic and/or molecular subtype signatures [3, 8–13]. For example, Wright et al. identified a 27-gene predictor to diagnose clinical distinct subtype of DLBCL [3]. Cai et al. built an expression-based signature incorporating up to 35 genes for both subtype classification and survival prediction [8]. These genes represented diverse biological roles involved in focal adhesion, cell cycle and Wnt signaling pathway.

Long non-coding RNAs (lncRNAs) are a recently discovered major class of non-coding RNAs (ncRNAs) with more than 200 nucleotides in length [14]. A large number of studies have suggested that lncRNAs function as key regulatory player in a broad range of biological processes, including cell differentiation, development [15]. The dysregulation of lncRNAs has been strongly associated with tumorigenesis, tumor progression and metastasis, highlighting the emerging roles of lncRNAs as diagnostic and prognostic biomarkers as well as potential therapeutic targets in a variety of cancer types [16, 17]. There is growing evidence that cancer subtype could be characterized by differentiated lncRNA expression pattern, suggesting the potential of lncRNAs as potent biomarkers in cancer subtype. Several studies have observed subtype-specific lncRNA expression pattern between lung adenocarcinoma and squamous cell carcinoma [18, 19]. In breast cancer, the correlation between lncRNA expression and tumor subtype has also been investigated and some subtype-specific lncRNAs were identified [19–21]. For example, a well-known lncRNA *HOTAIR* was up-regulated in the HER2-enriched subgroup [20]. Our previous work has indicated the prognostic roles of lncRNAs in DLBCL patients [22]. Furthermore, recent studies demonstrated that lncRNA expression patterns can characterize distinct stages of B-cell development and activation [23, 24]. However, the expression pattern and clinical implication of lncRNAs between GCB and ABC DLBCL remain unclear.

In this study, we performed genome-wide comparative analysis of lncRNA expression profiles and investigated differentiated lncRNA expression pattern between GCB and ABC DLBCL. By applying the weighted voting algorithm, we identified a panel of 17 lncRNA biomarkers that are able to discriminate GCB and ABC subtypes with high performance. Furthermore, GCB-like and ABC-like subgroups defined by the lncRNA signature have a significantly different clinical outcome. The reproducible predictive power of 17-lncRNA signature was validated in other two independent DLBCL cohorts. In addition, an integrative analysis of lncRNA and mRNA was performed to infer functional roles of lncRNA biomarkers.

## Methods

### Patients’ samples

Gene expression microarray data and clinical information for DLBCL were downloaded from the Gene Expression Omnibus (GEO) database. Affymetrix gene expression profiles were performed using Affymetrix Human Genome U133 Plus 2.0 (HG-U133 Plus_2.0) for 2 cohorts of patients (GSE31312 and GSE10846) and using Affymetrix Human Genome U133A Array (HG-U133A) for 1 cohort of patients (GSE4475). After removing patients with no clinical or subtype information, a total of 905 DLBCL patients were included in our study (Table 1), comprising 426 patients from Visco’s study (the accession number is GSE31312) [10], 350 patients from Lenz’s study (the accession number is GSE10846) [25] and 129 patients from Hummel’s study (the accession number is GSE4475) [26].

**Table 1:** Clinical and pathological characteristics of patients with DLBCL in our study

Characteristics	Discovery cohort	Internal validation cohort	GSE31312 cohort	GSE10846 cohort	GSE4475 cohort
No. of patients	213	213	426	350	129
Age, year					
>60	121	123	244	196	72
≤60	92	90	182	154	57
Gender					
Female	101	82	183	152	54
Male	112	131	243	184	74
Unknown				14	1
Stage					
I/II	97	106	203	160	36
III/IV	116	107	223	184	48
Unknown				6	45
No. of extranodal sites					
<2	167	170	337	299	
≥2	46	43	89	26	
Unknown				25	
LDH					
0	72	61	133	140	
1	120	133	253	156	
Unknown	21	19	40	54	
ECOG					
<2	168	171	339	256	
≥2	45	42	87	74	
Unknown				20	
Subtype					
GCB	106	121	227	183	74
ABC	107	92	199	167	55
Unclassified					
Survival status					
Dead	80	74	154	143	51
Alive	133	139	272	207	42
Unknown					36

### Acquisition and analysis of lncRNA expression profiles

Raw CEL files of three independent patient cohorts were downloaded from the GEO database. The raw array data were uniformly pre-processed and normalized using the robust multi-array average (RMA) algorithm [27]. After background correction, quantile normalization and log2-transformation, the z-score transformation was applied for scaling expression intensities of each probe [28].

The probe annotation sequences of HG-U133 Plus_2.0 and HG-U133A were obtained from the Affymetrix website (http://www.affymetrix.com/estore/). Then probe sequences were re-mapped to the human genome (GRCh38) and lncRNA genes derived from GENCODE (release 21) using SeqMap tool [29]. Those probes that were uniquely mapped to the human genome and lncRNA genes with no mismatch were retained for further analysis. Finally, 3215 (covering 2330 lncRNAs for HG-U133 Plus_2.0) and 855 (covering 663 lncRNAs for HG-U133A) lncRNA-specific probes were obtained by cross-referencing the chromosomal position of probes and the chromosomal position of lncRNA genes according to previous studies [30–32]. For those lncRNAs with multiple probes, the expression values of lncRNAs were produced by using the mean value of multiple probes.

### Statistical analysis for subtype classification and prognosis prediction

#### Analysis of lncRNA expression profiles

The unpaired two-tailed Student’s *t*-test was used to determine the statistically significant difference in lncRNA expression between ABC and GCB subgroups. The method of false discovery rate (FDR) defined by Benjamini and Hochberg [33] was used for multiple testing correction. Those lncRNAs with *t*-test p-value <0.01 and FDR < 0.15 were identified as differentially expressed lncRNAs between ABC and GCB subgroups. Unsupervised hierarchical clustering of both DLBCL patients and lncRNAs was performed with R software using the euclidean distance and complete linkage method.

#### Formulation of lncRNA-based molecular signature

To construct a lncRNA-based molecular signature for subtype classification and prognosis prediction, we developed a supervised subgroup predictive classifier using the weighted voting algorithm as previously described [34] based on the weighted votes of a set of informative lncRNAs. The weighted votes was defined as *W*_*L*_ *V*_*L*_, where *W*_*L*_ is a weighting factor that measures how well this lncRNA is correlated with the subgroup classification and was calculated as *w*_*L*_ = (*μ*_*ABC*_ − *μ*_*GCB*_)/(*σ*_*ABC*_ − *σ*_*GCB*_), and *V*_*L*_ represents the deviation of the expression level of this lncRNA in the sample from the decision boundaries between the subgroup means and was calculated as ${v}_L=\Big|{e}_L-\left(\mu {}_{ABC}+{\mu}_{GCB}\right)/2\Big|$. Finally, for a given test sample, the weighted votes of informative lncRNAs for each subgroup was summed to form a final total votes *V*_*ABC*_ and *V*_*GCB*_, and this given sample was assigned to the winning subgroup with the higher final total votes.

#### Identification of lncRNA biomarkers associated with clinically molecular subtype and prognosis

To obtain an optimal lncRNA molecular signature for subtype classification and prognosis prediction, the above-mentioned supervised predictive classifier was constructed with different numbers of differentially expressed lncRNAs using 5-fold cross-validation strategy and 100 randomized permutations. The average number of misclassified patients of 100 randomized permutations for predictive classifier constructed by a specific number of lncRNAs (*n* = 1, 2, 3, ……, 156) as follows: $averag{e}_{errorN}=\left({\displaystyle \underset{i=1}{\overset{100}{\varSigma }}}{\displaystyle \underset{j=1}{\overset{5}{\varSigma }}} error\right)/100$. The number of lncRNAs with a balance between classification accuracy and number was chosen as the optimal number *k*. The frequencies of lncRNAs in 500 candidate lncRNA ranking list according to their signal-to-noise ratio were ranked and top *k* of the ranked lncRNAs was identified as lncRNAs biomarkers which were used to derive an optimal lncRNA molecular signature using the weighted voting algorithm for subtype classification and prognosis prediction.

### Survival analysis

The difference in overall survival and progression-free survival between the predicted subgroups of patients was plotted using the Kaplan-Meier curves method and was tested by the log-rank test. Univariate and multivariate Cox regression analysis were performed to evaluate the association between the lncRNA-based molecular signature and survival with and without other clinical variables in each dataset. Hazard ratios (HR) and 95% confidence intervals (CI) were calculated by Cox proportional hazards regression model. All these statistical analyses were conducted using the R package and Bioconductor.

### Functional enrichment analysis

The functional enrichment analysis of Gene Ontology (GO) and Kyoto encyclopedia of genes and genomes (KEGG) was conducted using DAVID Bioinformatics Tool (https://david.ncifcrf.gov/, version 6.7) [35] to identify significantly enriched biological themes including GO terms and KEGG pathways. GO functional terms limited in the “Biological Process” (GOTERM-BP-FAT) and KEGG pathways with FDR <0.05 were considered significant.

## Results

### Identification of lncRNA biomarkers associated with clinically molecular subtype

Here, 426 DLBCL patients from the GSE31312 cohort, which is the largest patient dataset, were randomly assigned to a discovery cohort (*n* = 213) and an internal validation cohort (*n* = 213). We first compared the lncRNAs expression profiles and determined altered lncRNA expression associated with clinically molecular subtype in the discovery cohort. In total, 156 lncRNAs were differentially expressed between the two major clinically molecular subtypes of DLBCL (ABC and GCB) using the unpaired two-tailed Student’s *t*-test with *p*-value <0.01 and FDR < 0.15 (Additional file 1: Table S1). Among the differentially expressed lncRNAs, 56 lncRNAs were up-regulated in the ABC subgroup and 100 lncRNAs were up-regulated in the GCB subgroup. These differentially expressed lncRNAs were considered as candidate lncRNAs biomarkers associated with clinically molecular subtype.

**Fig. 1:**
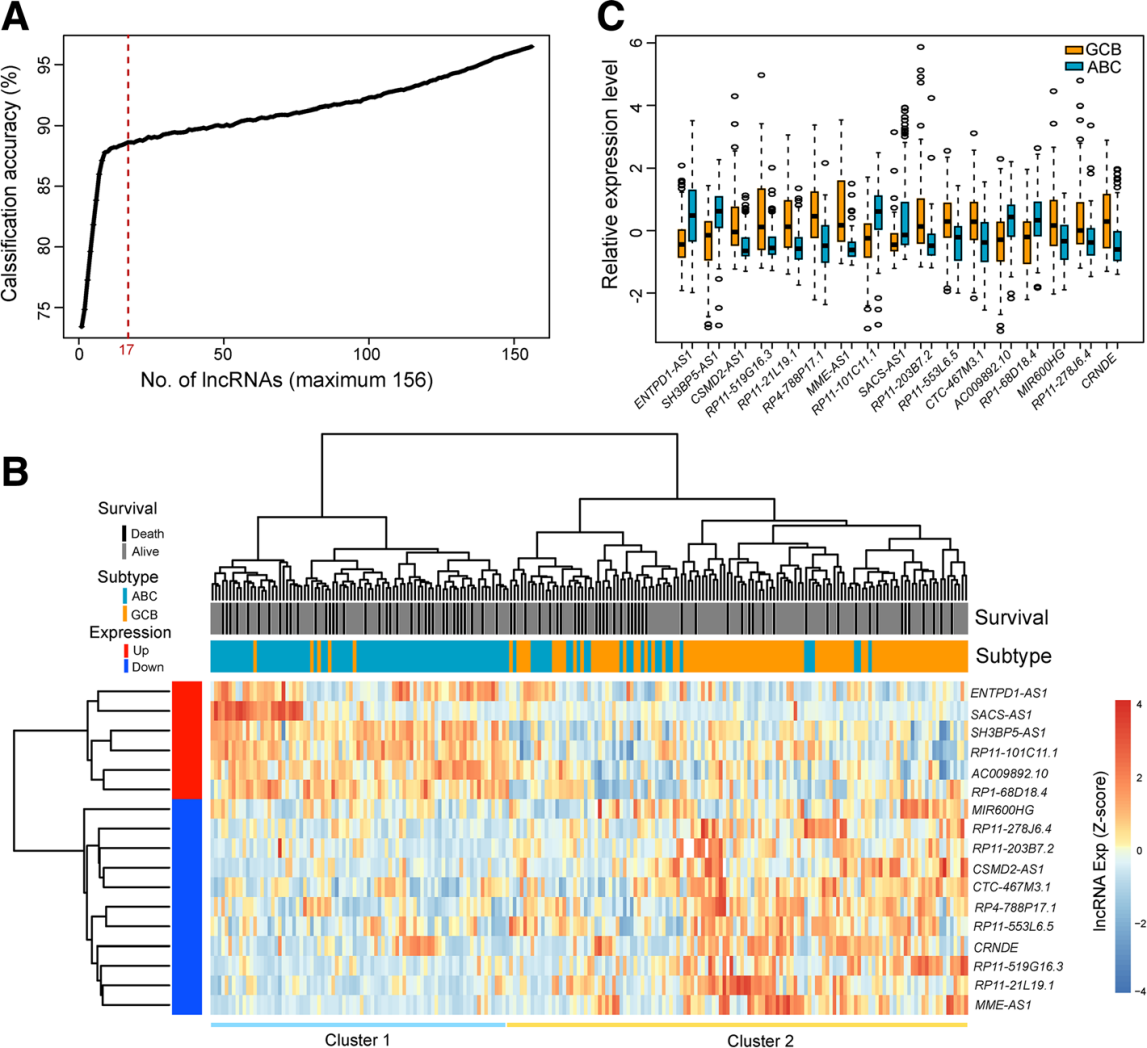
Identification of subtype-specific lncRNA biomarkers in the discovery cohort. **a** The classification accuracy for top *K*-lncRNA model using 5-fold cross-validation strategy and 100 randomized permutations. **b** The unsupervised hierarchical clustering heatmap of 213 patients based on selected optimal 17 lncRNAs biomarkers. **c** Expression patterns of selected optimal 17 lncRNAs biomarkers in the GCB and ABC subtypes

To identify optimal lncRNA biomarkers associated with clinically molecular subtype, we selected specific number of differentially expressed lncRNAs (number = 1, 2, 3, …, 156) to develop the supervised subtype predictive classifier using the weighted voting algorithm for distinguishing ABC and GCB DLBCL samples. The average number of misclassified samples in the 5-fold cross-validation analysis with 100 permutations was calculated and the accuracy of subgroup classifier was plotted (Fig. 1a) when increasing numbers of differentially expressed lncRNAs in the subgroup classifier. We found that 17 lncRNAs could yield a balance between classification accuracy and the number of lncRNAs. When choosing more than 17 lncRNAs, there is a decrease or very slight increase in prediction accuracy (Fig. 1a). Therefore, top 17 of the ranked lncRNAs according to their signal-to-noise ratio were identified as optimal lncRNA biomarkers (Table 2).

**Table 2:** Candidate lncRNAs biomarkers associated with clinically molecular subtype and prognosis of DLBCL

Ensembl id	Gene symbol	Chromosomal position	*p*-value	FDR	signal-to-noise ratio
ENSG00000226688.5	ENTPD1-AS1	Chr 10: 95,753,206-96,090,238 (−)	5.34E-10	1.78E-07	0.453
ENSG00000229558.2	SACS-AS1	Chr 13: 23,418,971-23,428,869 (+)	2.2E-07	3.94E-05	0.404
ENSG00000224660.1	SH3BP5-AS1	Chr 3: 15,254,184-15,264,493 (+)	4.93E-12	3.83E-09	0.502
ENSG00000231090.1	RP11-101C11.1	Chr 1: 55,217,861-55,234,177 (+)	3.88E-09	1.13E-06	0.421
ENSG00000224730.1	AC009892.10	Chr 19: 54,635,722-54,638,892 (−)	1.03E-07	2.36E-05	0.38
ENSG00000255443.1	RP1-68D18.4	Chr 11: 35,210,343-35,214,985 (−)	3.48E-07	5.8E-05	0.361
ENSG00000236901.4	MIR600HG	Chr 9: 123,109,494-123,115,477 (−)	9.02E-07	1.4E-04	0.359
ENSG00000279130.1	RP11-278 J6.4	Chr 5: 143,406,959-143,407,420 (+)	2.57E-06	3.737E-04	0.341
ENSG00000260303.1	RP11-203B7.2	Chr 4: 146,052,604-146,056,762 (−)	1.33E-07	2.57E-05	0.395
ENSG00000231163.4	CSMD2-AS1	Chr 1: 33,868,953-33,885,458 (+)	2.76E-10	1.29E-07	0.493
ENSG00000245864.2	CTC-467 M3.1	Chr 5: 88,676,218-88,722,831 (+)	1.12E-07	2.36E-05	0.379
ENSG00000223479.3	RP4-788P17.1	Chr 1: 73,635,216-73,715,214 (+)	2.91E-12	3.39E-09	0.514
ENSG00000259976.1	RP11-553 L6.5	Chr 3: 114,314,501-114,316,179 (−)	6.09E-08	1.58E-05	0.386
ENSG00000245694.7	CRNDE	Chr 16: 54,918,863-54,929,189 (−)	3.49E-06	4.71E-04	0.328
ENSG00000259354.4	RP11-519G16.3	Chr 15: 45,448,427-45,513,767 (+)	3.7E-10	1.44E-07	0.494
ENSG00000254418.1	RP11-21 L19.1	Chr 11: 14,262,846-14,273,691 (−)	2.96E-11	1.73E-08	0.507
ENSG00000240666.2	MME-AS1	Chr 3: 155,158,370-155,183,285 (−)	4.33E-15	1.01E-11	0.666

To investigate the expression pattern of 17 optimal lncRNA biomarkers associated with clinically molecular subtype, we clustered 213 DLBCL samples in the discovery cohort according to the expression levels of 17 optimal lncRNA biomarkers by hierarchical clustering analysis. As shown in Fig. 1b, 213 DLBCL samples in the discovery cohort were separated into two distinctive patient subgroups which were highly correlated with clinically molecular subtype (*p* < 0.001, Chi-square test; Fig. 1b). The left branch (Cluster 1) contained the majority of ABC-DLBCL patients (79/107; 73.8%) and the right branch (Cluster 2) contained close to all of GCB-DLBCL patients (101/106; 95.3%). These two distinctive patient subgroups were both well characterized by the expression patterns of 17 lncRNA biomarkers in which 6 lncRNAs were up-regulated and 11 lncRNAs were down-regulated in patients included in Cluster 1 relative to those included in Cluster 2 (Fig. 1c). The above results demonstrated that these 17 lncRNA biomarkers might have a predictive power in the subtype classification of DLBCL patients.

### A lncRNA-based molecular signature for subtype classification and prognosis prediction in DLBCL patients

**Fig. 2:**
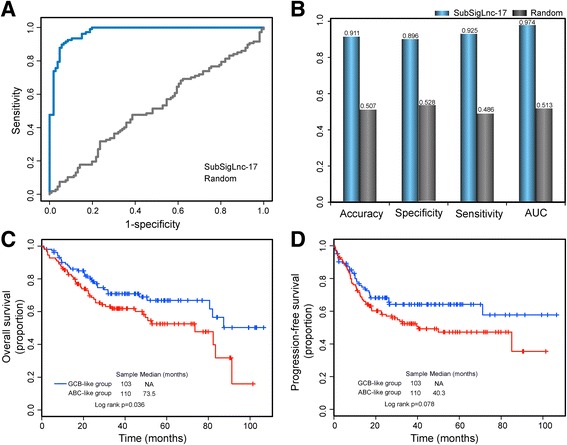
Performance evaluation of SubSigLnc-17 in the subtype classification and prognosis for DLBCL patients in the discovery cohort. **a** ROC analysis of the sensitivity and specificity of subtype prediction by the SubSigLnc-17. **b** Performance comparison in subtype prediction between SubSigLnc-17 and random lncRNAs. **c** Kaplan-Meier survival curves of overall survival between predicted GCB-like group and ABC-like group by SubSigLnc-17. **d** Kaplan-Meier survival curves of progression-free survival between predicted GCB-like group and ABC-like group by SubSigLnc-17

Since these 17 lncRNA biomarkers exhibited better ability in subtype classification, we integrated these 17 lncRNA biomarkers to derive a lncRNA-based molecular signature (hereafter inferred as SubSigLnc-17) and constructed a supervised subgroup predictive classifier using the weighted voting algorithm based on the expression patterns of SubSigLnc-17 for predicting molecular subtype and outcome. The SubSigLnc-17 was able to assign a DLBCL patient in the discovery cohort into ABC or GCB subgroups when the probability of this patient belonging to the ABC or GCB subgroups is greater than 50%. As a result, the SubSigLnc-17 performed very well on the discovery cohort and achieved a very high AUC of 0.974 with a specificity of 89.6% and a sensitivity of 92.5% (Fig. 2a and b). The SubSigLnc-17 correctly classified 99 out of 107 ABC DLBCL patients and 95 out of 106 GCB DLBCL patients with an accuracy of 91.1% (Fig. 2b). Moreover, the Kaplan-Meier analysis for overall survival and progression-free survival demonstrated significant differences between the two predicted subgroups by the SubSigLnc-17 (*p* = 0.036 for overall survival and *p* = 0.078 for progression-free survival, respectively, log-rank test; Fig. 2c and d). The 5-year overall survival of DLBCL patients in the predicted GCB-like group was 66.8%, whereas the corresponding rate in the predicted ABC-like group was 52.5%. The hazard ratios of predicted ABC-like group versus GCB-like group for overall survival was 1.614 in the univariate analysis (95% CI 1.029 to 2.532, *p* = 0.037), indicating that the SubSigLnc-17 has a significant association not only with molecular subtype but also with prognosis.

### Further validation of lncRNA-based molecular signature in the internal validation cohort and entire GSE31312 cohort

**Fig. 3:**
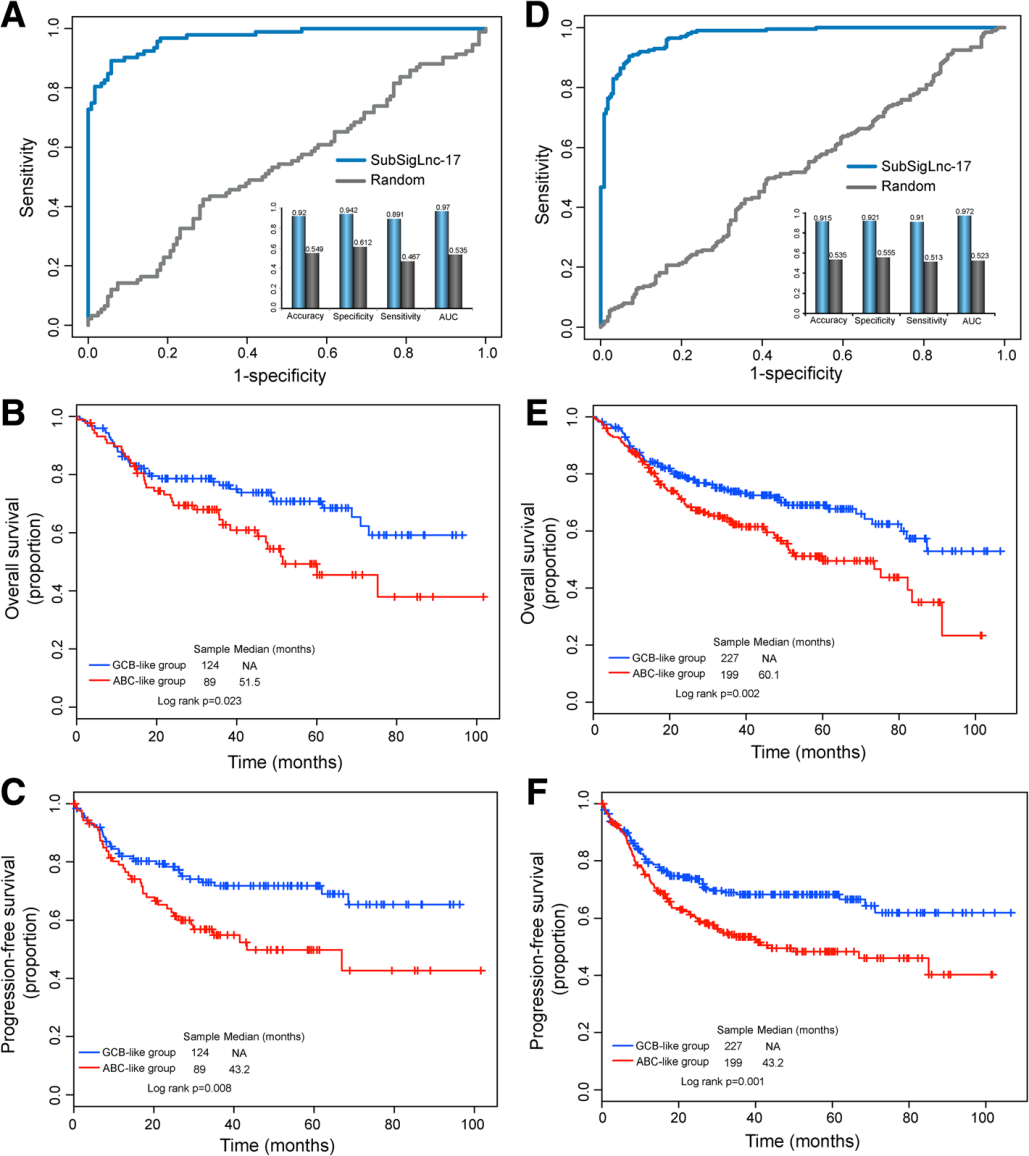
Validation of SubSigLnc-17 in the subtype classification and prognosis for DLBCL patients in the internal validation cohort and entire GSE31312 cohort. ROC analysis of the sensitivity and specificity of subtype prediction by the SubSigLnc-17 in the **a** internal validation cohort and **d** entire GSE31312 cohort. Kaplan-Meier survival curves of overall survival between predicted GCB-like group and ABC-like group by SubSigLnc-17 in the **b** internal validation cohort and **e** entire GSE31312 cohort. Kaplan-Meier survival curves of progression-free survival between predicted GCB-like group and ABC-like group by SubSigLnc-17 in the **c** internal validation cohort and **f** entire GSE31312 cohort

Further validation of the predictive power of SubSigLnc-17 in subtype classification and prognosis prediction was carried out using the internal validation cohort and entire GSE3132 cohort. The result of internal validation cohort indicated that the SubSigLnc-17 could distinguish ABC and GCB DLBCL patients with an AUC of 0.97 (Fig. 3a). The SubSigLnc-17 correctly classified 82 out of 92 ABC DLBCL patients and 114 out of 121 GCB DLBCL patients with an accuracy of 92%, a specificity of 94.2% and a sensitivity of 89.1%. In the predicted ABC-like group, the overall survival rate was significantly lower than that in the predicted GCB-like group (*p* = 0.023, log-rank test; Fig. 3b), and the 5-year overall survival rates of patients in the predicted ABC-like group and in the predicted GCB-like group were 49.3% and 70.9%. Moreover, progression-free survival was also significantly different between the predicted two subtype groups (*p* = 0.008, log-rank test; Fig. 3c), and patients in the predicted ABC-like group experienced a lower rate of progression-free survival after 5 years (49.8% vs. 71.8%). The univariate analysis revealed that the SubSigLnc-17 was still significantly associated with overall survival in the internal validation cohort (HR = 1.695, 95% CI 1.072 to 2.682, *p* = 0.024).

Similar results were observed when the SubSigLnc-17 was tested in the entire GSE31312 cohort, which resulted in an AUC of 97.2% with a specificity of 92.1% and a sensitivity of 91% (Fig. 3d). Among 426 DLBCL patients in the entire GSE31312 cohort, 390 patients (209 out of 227 GCB patients and 181 out of 199 ABC patients) were assigned to the corresponding subtype groups by the SubSigLnc-17 with an accuracy of 91.5%. Moreover, there was a significant difference in overall survival and progression-free survival between the two predicted patient subgroups (*p* = 0.002 for overall survival and *p* = 0.001 for progression-free survival, respectively, log-rank test; Fig. 3e and f). The 5-year overall survival and progression-free survival rates of DLBCL patients in the predicted GCB-like group were 69.1% and 68.3%, whereas the corresponding rate in the predicted ABC-like group was 51.1% and 48.3%. The hazard ratios of predicted ABC-like group versus GCB-like group for overall survival was 1.638 in the univariate analysis (95% CI 1.19 to 2.254, *p* = 0.002; Table 3).

**Table 3:** Univariate and multivariate Cox regression analysis of overall survival in each dataset

Variables	Univariate analysis			Multivariate analysis		
	HR	95% CI of HR	*P* value	HR	95% CI of HR	*P* value
GSE31312 cohort (*n* = 426)						
SubSigLnc-17 (ABC vs. GCB)	1.638	1.19-2.254	0.002	1.422	0.997-2.028	0.052
Age (> = 60 vs. <60)	2.01	1.41-2.864	1.12E-04	1.946	1.315-2.881	8.79E-04
Gender (Male vs. Female)	0.959	0.697-1.32	0.798	0.843	0.597-1.189	0.331
Stage (III/IV vs. I/II)	2.314	1.646-3.251	1.35E-06	1.707	1.135-2.567	0.01
LDH (High vs. Normal)	2.035	1.362-3.04	5.19 E-04	1.475	0.973-2.236	0.067
No. of extranodal sites (≥2 vs. < 2)	2.247	1.598-3.16	3.23E-06	1.778	1.213-2.605	0.003
ECOG (≥2 vs. < 2)	2.195	1.556-3.097	7.48E-06	1.584	1.065-2.355	0.023
GSE10846 cohort (*n* = 350)						
SubSigLnc-17 (ABC vs. GCB)	2.364	1.673-3.341	1.10E-06	2.093	1.391-3.149	3.94E-04
Age (> = 60 vs. <60)	2.099	1.464-3.009	5.50E-05	1.988	1.31-3.016	0.001
Gender (Male vs. Female)	1.017	0.724-1.429	0.922	0.993	0.676-1.46	0.972
Stage (III/IV vs. I/II)	1.747	1.239-2.464	0.001	1.147	0.762-1.727	0.51
LDH (High vs. Normal)	2.643	1.791-3.899	9.72E-07	2.038	1.341-3.096	8.59E-04
No. of extranodal sites (≥2 vs. < 2)	1.899	1.087-3.317	0.024	1.183	0.58-2.415	0.644
ECOG (≥2 vs. < 2)	2.968	2.091-4.214	1.19E-09	1.907	1.246-2.918	0.003

### Confirmation of predictive power of lncRNA-based molecular signature using two independent DLBCL patient cohorts with a different platform

**Fig. 4:**
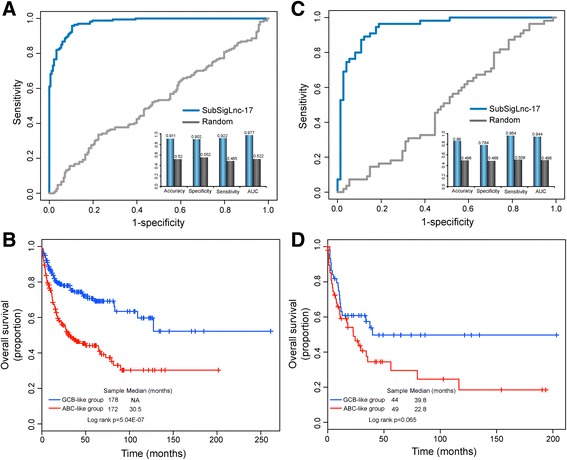
Independent validation of SubSigLnc-17 for prognosis prediction in two additional independent cohorts. Performance evaluation of SubSigLnc-17 in the **a** GSE10846 cohort and **c** GSE4475 cohort. Kaplan-Meier survival curves of overall survival between predicted GCB-like group and ABC-like group by SubSigLnc-17 in the **b** GSE10846 cohort and **d** GSE4475 cohort

To further test the robustness of the SubSigLnc-17, we examined the discriminatory power of the SubSigLnc-17 using two completely independent non-overlapped cohorts of 350 DLBCL patients obtained from Lenz’s study (the accession number is GSE10846) [25] and 129 patients obtained from Hummel’s study (the accession number is GSE4475) [26]. The SubSigLnc-17 was again shown capable of distinguishing ABC and GCB DLBCL patients in the GSE10846 cohort. The SubSigLnc-17 correctly classified 91.1% of patients (165 out of 183 GCB patients and 154 out of 167 ABC patients) into the corresponding subtype groups and achieved an AUC of 97.7% with a specificity of 90.2% and a sensitivity of 92.2% (Fig. 4a). Subgroups of patients characterized by the SubSigLnc-17 demonstrated different outcome. Overall survival was significantly better in the predicted GCB-like subgroup as compared with the predicted ABC-like subgroup, showing 5-year overall survival in 69.2% and 44.1% of patients in the predicted GCB-like and ABC-like subgroups, respectively (*p* = 5.04E-07, log-rank test; Fig. 4b).

Another independent DLBCL patient cohort (GSE4475), comprising of 129 patients, was based on a different Affymetrix microarray platform (HG-U133A). Therefore, we re-annotated the probes of Affymetrix HG-U133A as described in Methods and found that only 9 of 17 lncRNAs in the SubSigLnc-17 were covered on the Affymetrix HG-U133A array. Therefore, the SubSigLnc-17 only based on these 9 lncRNAs without re-estimating parameters was used to classify 129 DLBCL patients of GSE4475 into distinct patient subgroups. As shown in Fig. 4c, the SubSigLnc-17 represented by 9 lncRNAs for ABC and GCB discrimination achieved an AUC of 94.4% with accuracy of 86%, specificity of 78.4% and sensitivity of 96.4%, despite the fact that 8 lncRNAs in the SubSigLnc-17 based on Affymetrix HG-U133 Plus_2.0 is missing in the GSE4475 cohort based on Affymetrix HG-U133A which might reduce the predictive performance of the SubSigLnc-17. The Kaplan-Meier survival curves for the predicted ABC and GCB subgroups in the independent external GSE4475 also were marginally significantly different (*p* = 0.065, log-rank test; Fig. 4d). DLBCL patients assigned to the ABC subgroup tended to have shorter overall survival than those assigned to the GCB subgroup (median survival 22.8 months vs. 39.8 months). The respective absolute difference in 5-year overall survival rates between the predicted ABC and GCB subgroups was 20.2% (29.5% vs. 49.7%) for the GSE4475 cohort. In the univariate Cox regression model, the SubSigLnc-17 again maintained a significant or marginally significant correlation with overall survival in both GSE10846 cohort (HR = 2.364, 95% CI 1.673 to 3.341, *p* = 1.10E-06) and GSE4475 cohort (HR = 1.686, 95% CI 0.96 to 2.96, *p* = 0.069) .

### Independence of prognostic value of lncRNA-based molecular signature from other clinical factors

**Fig. 5:**
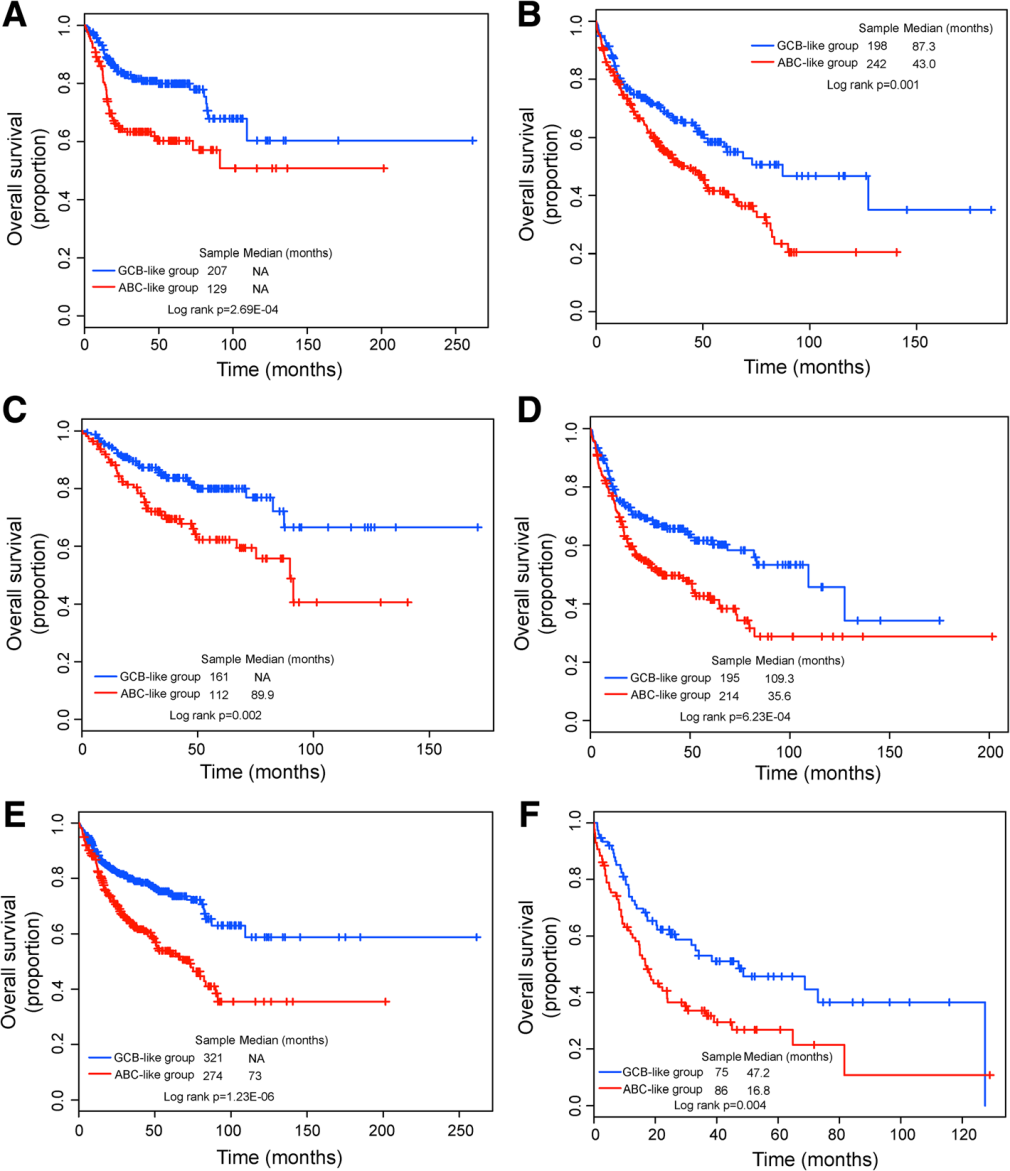
Prognosis prediction in patients stratified by age, LDH level and ECOG performance status. Kaplan-Meier survival curves of overall survival between predicted GCB-like group and ABC-like group by SubSigLnc-17 in the **a** younger group, **b** older group. **c** LDH < 1*normal group, **d** LDH > =1*normal group, **e** a good general health status group and **f** a poor general health status group

To investigate whether the prognostic value of the SubSigLnc-17 was independent of other clinical factors, we first performed multivariate Cox regression analyses using the following factors as categorical variables: the SubSigLnc-17 (ABC-like vs. GCB-like), age (≥60 vs. <60), gender (male vs. female), stage (III/IV vs. I/II), lactate dehydrogenase (LDH) level (high vs. normal), number of extranodal sites (≥2 vs. < 2) and Eastern cooperative Oncology Group (ECOG) performance status (≥2 vs. < 2). The results of multivariate analysis revealed that the SubSigLnc-17 was consistently associated with the outcome of patients with DLBCL after adjustment for other clinical variables in the GSE31312 and GSE10846 cohorts (HR = 1.422, 95% CI 0.997 to 2.028, *p* = 0.052 for GSE31312 cohort and HR = 2.093, 95% CI 1.391 to 3.149, *p* = 3.94E-04 for GSE10846; log-rank test) (Table 3). However, three clinical variables (including age, LDH and ECOG) were also found to be significantly correlated with patients’ overall survival. Therefore, we conducted the stratification analysis for these significant clinical variables to test whether the SubSigLnc-17 could provide additional prognostic value within the same clinical factors. For age alone, 776 DLBCL patients of the combined patient cohort (GSE31312 and GSE10846) were stratified into the younger group with ages below 60 years (*n* = 336) and the older group with above ages 60 years (*n* = 440). With the SubSigLnc-17, patients in the younger group were divided into ABC-like group and GCB-like group with significantly different survival (*p* = 2.69E-04, log-rank test) (Fig. 5a). The similar prognostic power of the SubSigLnc-17 was found in the older group in which patients with above ages 60 years were classified as either ABC-like with poor outcome (median survival 43 months) and GCB-like with good outcome (median survival 87.3 months) (Fig. 5b). Stratification analyses were repeated in patients with normal or high LDH level and revealed a statistically significant difference in overall survival between ABC-like and GCB-like groups in the patient subgroup stratified by LDH level. The predicted GCB-like patients had significantly better survival relative to predicted ABC-like patients in either subgroup of patients with LDH < 1*normal (not reach median survival vs. 89.9 months, *p* = 0.002, log-rank test) (Fig. 5c) or subgroup with LDH > =1*normal (median survival 109.3 months vs. 35.6 months, *p* = 6.23E-04, log-rank test) (Fig. 5d). Finally, the prognostic value of the SubSigLnc-17 for the patients with good or poor general health status was also assessed. 756 patients with ECOG information was stratified into a good general health status stratum (with ECOG performance status score < 2) (*n* = 595) and a poor general health status stratum (ECOG performance status score of 2 or greater) (*n* = 161). Survival analysis revealed that within each ECOG stratum, the SubSigLnc-17 was able to distinguish patients with significantly different survival despite having the same health status. For instance, among patients with ECOG performance status score < 2, the SubSigLnc-17 could further classify patients into the ABC-like group with the overall survival of 73 months and the GCB-like group with not reached median overall survival (*p* = 1.23E-06, log-rank test) (Fig. 5e). The similar prognostic value was observed in the subgroup of patients with ECOG performance status score of 2 or greater, results of separate series see Fig. 5f in which GCB-like patients have better overall survival than ABC-like patients (median survival 47.2 months vs. 16.8 months, *p* = 0.004, log-rank test). These results demonstrated that the SubSigLnc-17 was a significant independent predictor of prognosis and could provide additional prognostic value beyond conventional clinical factors.

## Discussion

DLBCL is mainly composed of GCB and ABC subtypes with distinct biological features and clinical implication. With the development of high-throughput technology, molecular heterogeneities between GCB and ABC subtypes have been well characterized at the mRNA and miRNA levels, and some subtype-specific mRNAs or miRNAs have been identified [8–10]. In recent years, the study of lncRNAs has given renewed impetus to cancer biology. The dysregulated lncRNA expression has been implicated in the initiation and progression of cancer [36]. Specifically, lncRNAs showed more tissue-, cell type- and cancer-specific expression patterns than protein-coding genes and miRNAs leading to the possibilities in better deciphering molecular heterogeneity of cancer subtypes [36, 37]. LncRNA expression profiles have been widely analyzed in several cancer subtypes, including gliomas [38], lung cancer [18, 19], colorectal cancer [39] and breast cancer [19, 20]. However, comprehensive characterization of lncRNA expression in DLBCL subtypes has not been performed.

As an initial step toward understanding lncRNA-level molecular disparity in DLBCL subtypes, we obtained and analyzed lncRNA expression profiles of 905 DLBCL patients using probe repurposing-based lncRNA-mining approach. By first separating DLBCL patients of the discovery cohort into either GCB or ABC subtypes based on their clinical information, we performed a comparative analysis for lncRNA expression pattern across GCB and ABC subtypes and uncovered 156 novel differentially expressed lncRNAs associated with either GCB or ABC subtypes. Several recent studies have shown that lncRNA were widely expressed during B-cell development and different lncRNAs played differential functional roles in distinct stages of B-cell development [23, 24]. Our finding has presented evidence that there was differentiated lncRNA expression pattern between GCB and ABC DLBCL, implicating that these subtype-specific lncRNAs may provide additional information for DLBCL subtype classification and prognosis. Therefore, we sought to investigate whether lncRNA expression can distinguish between GCB and ABC subtypes. By subjecting differentially expressed lncRNAs into the weighted voting algorithm, we identified 17 lncRNA biomarkers that are significantly associated with clinically molecular subtype. Results with unsupervised hierarchical clustering of 213 DLBCL patients in the discovery cohort confirmed the subtype-specific expression pattern of 17 lncRNA biomarkers. Considering the convenience of clinical use, these 17 lncRNA biomarkers were used to construct a weighted voting-based lncRNA molecular signature (termed SubSigLnc-17) which is able to distinguish between GCB and ABC subtypes with high performance. Moreover, subgroups of patients characterized by the SubSigLnc-17 signature demonstrated significantly different clinical outcome, indicating that the SubSigLnc-17 signature may include clinical implication about disease prognosis. These results suggested that lncRNA expression also can reflect characteristic of COO and have similar predictive ability for subtype classification and prognosis to those of mRNA or miRNA for DLBCL. The highly predictive power of the SubSigLnc-17 signature in subtype classification and prognosis for DLBCL was successfully validated through application in the internal validation cohort and another independent cohort as well as in the Hummel’s cohort with a different platform. These findings, together with our previous report that a six-lncRNA signature could also predict patients’ survival in DLBCL [22], demonstrated the important implication of lncRNA in DLBCL subtype classification and clinical outcome.

**Fig. 6:**
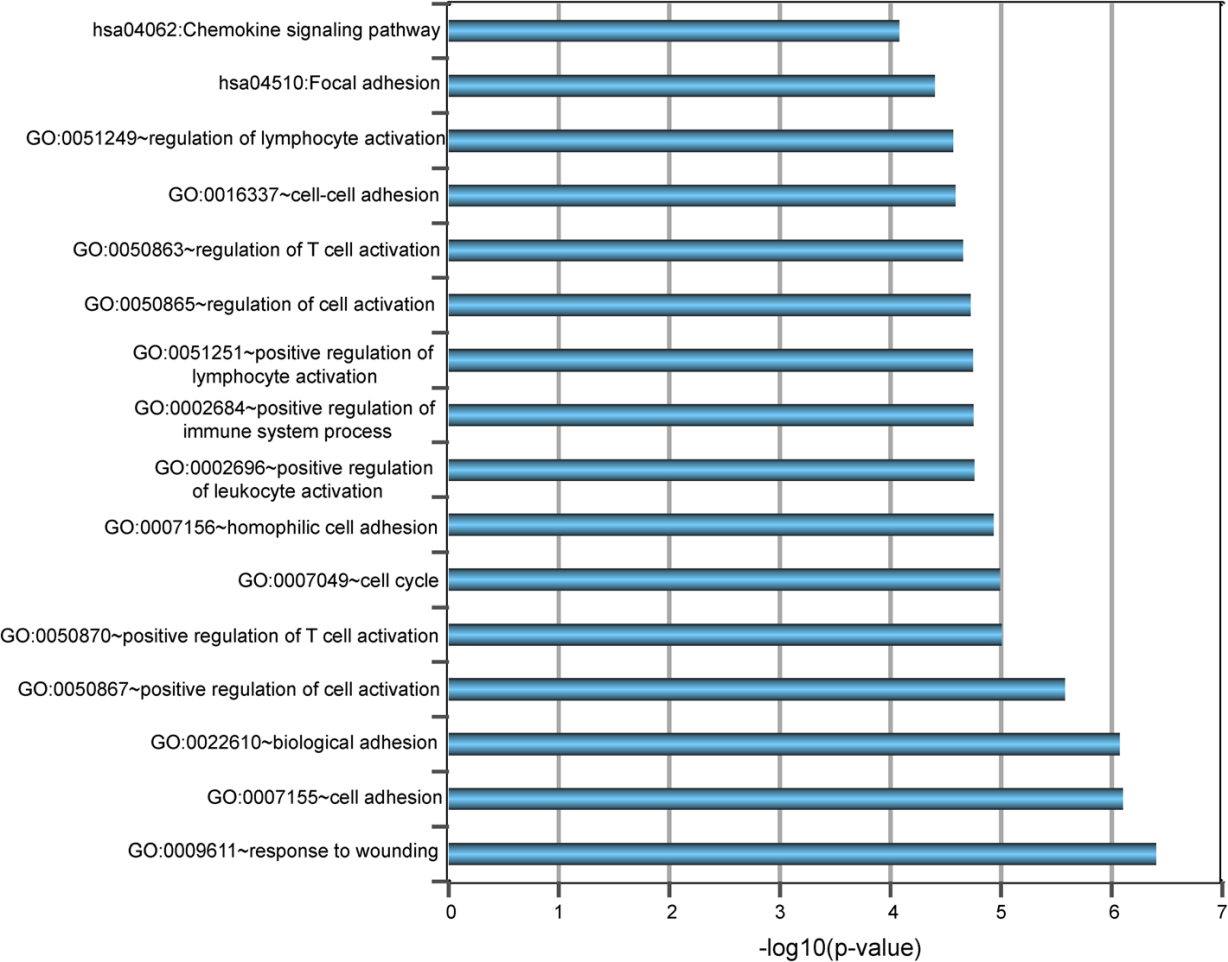
Results for GO and KEGG enrichment analysis

To gain more insights into the functional roles of the SubSigLnc-17 in DLBCL, we performed functional enrichment analysis for mRNAs co-expressed with 17 lncRNA biomarkers to investigate the associated biological processes and pathways. We firstly calculated the Pearson correlation coefficient of paired lncRNA and mRNA expression profiles of 426 patients in the GSE31312 cohort to measure the co-expressed relationships between 17 lncRNA biomarkers and mRNAs. Then mRNAs were ranked according to the Pearson correlation coefficient for each lncRNAs and the highest ranked mRNAs (top 0.5%) were selected as co-expressed mRNAs with lncRNA biomarkers. A total of 1206 mRNAs were positively correlated with at least one of 17 lncRNA biomarkers. In the GO analysis, 14 GO terms of biological process were significantly enriched among these mRNAs co-expressed with lncRNA biomarkers, including response to wounding, cell adhesion, T cell activation, cell cycle, leukocyte activation, immune system process and lymphocyte activation (Fig. 6). Furthermore, Focal adhesion and Chemokine signaling pathway also were found to be highly enriched in the KEGG pathway enrichment analysis. Taken together, GO and KEGG functional analysis demonstrated that 17 lncRNA biomarkers in SubSigLnc-17 significantly participated in immune- and cell cycle-associated biological processes.

## Conclusions

In summary, we reported a comprehensive comparative analysis of lncRNA expression pattern between GCB and ABC DLBCL, and identified several novel lncRNA biomarkers as indicators of subtype classification and prognosis in DLBCL. The underlying mechanisms whereby lncRNA biomarkers exerts their biological roles in immune-associated biological processes. With further prospective validation, our study will improve the understanding of underlying molecular heterogeneities in DLBCL and provide candidate lncRNA biomarkers in DLBCL classification and prognosis.

## Additional file

Additional file 1: Table S1. Differential expression of lncRNAs between the two major clinically molecular subtypes of DLBCL (ABC and GCB) (*p*-value < 0.01 and FDR < 0.15). (XLS 42 kb)

## Abbreviations

DLBCL: Diffuse large B-cell lymphoma
NHL: non-Hodgkin lymphoma
R-CHOP: Rituximab combined with traditional chemotherapy of cyclophosphamide-doxorubicin-vincristine-prednisone
COO: Cell of origin
GCB: Germinal center B-cell-like
ABC: Activated B-cell-like
PFS: Progression-free survival
LncRNAs: Long non-coding RNAs
NcRNAs: Non-coding RNAs
GEO: Gene expression omnibus
RMA: Robust multi-array average
FDR: Fa discovery rate
HR: Hazard ratios
CI: Confidence intervals
GO: Gene ontology
KEGG: Kyoto encyclopedia of genes and genomes
